# Stirring the motivational soup: within-person latent profiles of motivation in exercise

**DOI:** 10.1186/s12966-017-0464-4

**Published:** 2017-01-14

**Authors:** Magnus Lindwall, Andreas Ivarsson, Karin Weman-Josefsson, Linus Jonsson, Nikos Ntoumanis, Heather Patrick, Cecilie Thøgersen-Ntoumani, David Markland, Pedro Teixeira

**Affiliations:** 1 Department of Food and Nutrition, and Sport Science, University of Gothenburg, Gothenburg, Sweden; 2 Department of Psychology, University of Gothenburg, Gothenburg, Sweden; 3 Research on Welfare, Health and Sport, Halmstad University, Halmstad, Sweden; 4 School of Psychology & Speech Pathology, Curtin University, Bentley, Australia; 5 Envolve PeopleCare, Farmington, USA; 6 School of Sport, Health and Exercise Sciences, Bangor University, Bangor, UK; 7 Faculty of Human Kinetics, Technical University of Lisbon, Lisbon, Portugal

**Keywords:** Motivation profile, Person-oriented, Exercise, Need satisfaction, Self-determination

## Abstract

**Background:**

The purpose of the present study was to use a person-oriented analytical approach to identify latent motivational profiles, based on the different behavioural regulations for exercise, and to examine differences in satisfaction of basic psychological needs (competence, autonomy and relatedness) and exercise behaviour across these motivational profiles.

**Methods:**

Two samples, consisting of 1084 and 511 adults respectively, completed exercise-related measures of behavioural regulation and psychological need satisfaction as well as exercise behaviour. Latent profile analyses were used to identify motivational profiles.

**Results:**

Six profiles, representing different combinations of regulations for exercise, were found to best represent data in both samples. Some profiles were found in both samples (e.g., low motivation profile, self-determined motivation profile and self-determined with high introjected regulation profile), whereas others were unique to each sample. In line with the Self-Determination Theory, individuals belonging to more self-determined profiles demonstrated higher scores on need satisfaction.

**Conclusions:**

The results support the notions of motivation being a multidimensional construct and that people have different, sometimes competing, reasons for engaging in exercise. The benefits of using person-oriented analyses to examine within-person interactions of motivation and different regulations are discussed.

## Background

Investigating the underlying processes that give rise to different patterns in health behavior does not only constitute a key challenge in research but also in applied work [1]. One theory about motivation that has gained particularly strong interest among researchers in different areas such as sport and exercise psychology [2] is self-determination theory (SDT; [3, 4]). Instead of viewing motivation as a one-dimensional concept, focusing on quantity, in SDT motivation is conceptualized as multidimensional where different qualities of motivation are the key focus [5]. In the SDT sub-theory of organismic integration theory (OIT; [3]), there are three broad types of motivation: intrinsic motivation, extrinsic motivation and amotivation. Intrinsic motivation is based on interest and satisfaction inherent in engaging in an activity. Extrinsic motivation encompasses four forms of behavioral regulation which, in contrast to intrinsic motivation, focus on consequences that are separate from engaging in the activity itself. Integrated regulation relates to engaging in the activity because it is concordant with an individual’s other personal goals and values. Identified regulation is based on consciously valuing the outcomes of engaging in an activity. Introjected regulation is characterized by being motivated to engage in an activity to avoid feelings of guilt or to enhance one’s self-worth. External regulation concerns being motivated to obtain rewards or to avoid punishments administered by others. Finally, amotivation relates to a lack of intention to act. Intrinsic, integrated and identified regulations are considered relatively autonomous or self-determined forms of motivation, reflecting a sense of volition and personal causation with respect to a behavior. External and introjected regulations are considered relatively controlled forms of motivation, whereby behavior is governed by externally- or internally-imposed pressures to act. Within SDT, autonomous motivation is hypothesized to positively influence behavioral engagement and psychological well-being [6]. Many studies have provided support for this hypothesis within the context of exercise and health (for reviews, see [7–9]). For example, in a systematic review, Teixeira et al. [9] found consistent support for positive associations between autonomous forms of behavioral regulation and exercise behavior.

Another key theoretical tenet of SDT is that individuals have an innate and universal need to feel autonomous, competent and related [4]. Competence refers to feelings of effectance and capability, autonomy represents feelings of volition or self-determination while relatedness refers to feelings of social inclusion and closeness. In the SDT [4, 10] self-determined motivation will be promoted when the three needs are satisfied. This hypothesis has also been supported in a number of studies [7, 11]. The link between autonomous motivation, need satisfaction and health outcomes has also been supported in a meta-analysis of 184 independent data sets [8].

A central assumption of the OIT is that the motivational type varies along a continuum, ranging from controlled to self-determined types of regulation [12]. The assumption underlining the continuum, and the use of the continuum itself, has however lately been questioned in a number of papers from different perspectives [13–15]. For example, using Rasch analysis to evaluate the postulate that motivation is best represented along a continuum of relative autonomy, Chemolli and Gagne’ [14] found support for the notion that the different regulations should be described as varying in kind rather than in degree and that the empirical evidence points to multidimensional rather than unidimensional conceptualization of motivation. Gunnell and Gaudreau [15], further problematized the unidimensionality of the OIT continuum and argued for a bi-factor model where motivation is conceptualized as consisting of both a general factor of motivation and specific factors representing the different regulations with different motivational qualities.

Another central assumption, linked to the OIT, is that people are motivated by a variety of reasons at the same time, and that multiple types of regulations operate together, simultaneously, to create the overall motivational pattern of the individual [10, 16]. This idea of simultaneous, complex and dynamic interplay of different regulations at the same time *within the* individual, also being conceptualized as the “motivational soup” [17], has however rarely been empirically tested in the context of exercise behavior in natural settings, or in motivational science overall. Traditionally, researchers have analyzed each motivational regulation separately or created a combined score of the different regulations by calculating a relative autonomy index [18], thereby avoiding to use information from all regulations simultaneously to target questions of interactions effects among the regulations.

Typically previous studies have utilized a more traditional variable-oriented approach [19], where different variables (e.g., the different regulations) are related to other variables (e.g., exercise behavior) in a linear fashion and interpreted at a between-person level rather than at a within-person level. Thus, the typical conclusion of such studies is that individuals with a higher score, compared to the average, on for example identified regulation exercise more regularly, compared to the average. Although these are interesting findings, such results and analyses do not directly target the hypothesis suggested by several SDT scholars [3, 4, 10, 16, 20] of different types of motivation coexisting within the individual to different degrees and the potential interactions between different regulations. In general, using a traditional variable-oriented analytical approach when examining complex interactions may be less than optimal, in particular, if there are theoretical reasons to expect patterns of interactions between more than two variables within persons [19]. More specifically, variable-oriented analyses are less able to capture the hypothesized complex simultaneous interplay, or push and pull, of different regulations *within the person* and how these within-person patterns give raise to specific patterns of exercise behavior.

Recently researchers within the field of SDT and physical activity have started to adopt a more person-oriented approach, trying to identify different clusters of individuals based on their motivational profiles. Using a person-oriented approach in the analyses of data could provide a complementary and unique insight in the underlying patterns of motivational processes. Studies adopting a person-oriented approach have been carried out in different fields, such as education [21], sports [22], and physical education [23–26].

Few studies have targeted motivational profiles of physical activity or exercise in adult populations. In a study of 540 mainly physically active Japanese adults, Matsumoto and Takenaka [27] found four clusters: a self-determined cluster (high in intrinsic motivation and identified regulation), a moderate motivational profile (moderate values in all regulations), a non-self-determined profile (non-self-determined regulations being higher than self-determined regulations) and an amotivation profile (high in amotivation and low in all other types). Guerin and Fortier [28] examined profiles in 120 Canadian adults who failed to meet recommended levels of physical activity. They identified three clusters: the self-determined (highest scores on intrinsic, moderate scores on identified and low scores on external and introjected), the motivated (moderate scores on intrinsic and identified, highest scores on introjected and low scores on external) and a low-self-determined (low scores on intrinsic and identified regulation). Finally, Friedrichs and colleagues [29] also found three clusters in their large study using approximately 2500 relatively sedentary Dutch adults. The first cluster, consisting of individuals with high scores on autonomous motivation and low to moderate scores on controlled motivation, were labelled the autonomous motivation cluster. The second cluster, the controlled motivation cluster, included individuals being high on controlled motivation and moderate on autonomous motivation, whereas the third cluster, the low motivation cluster, reported low scores on autonomous motivation and low to moderate scores on controlled motivation.

In terms of examining motivation and exercise behavior in adults, we are however not aware of any study that has explored different motivational profiles in exercise and linked such profiles to basic need satisfaction and exercise. Consequently there seems to be a gap in the understanding of how different combinations of regulations for exercise within-persons create different motivational profiles in adults and how these profiles differ in terms of satisfaction of basic psychological needs (competence, autonomy and relatedness) and exercise behavior.

A potential methodological weakness in all the person-oriented studies referred to above is the use of cluster analyses when identifying the number of different clusters/profiles. Compared to more recent analytical approaches to person-oriented analyses such as latent profile analyses (LPA), cluster analyses have a number of weaknesses [30, 31]. For example, although LPA, like cluster analyses is exploratory in its nature, LPA is a model-based technique that offers more flexibility in terms of model specification. In fact, cluster analysis may be viewed as a very restricted form of LPA [31]. Further, LPA offers several fit indices, providing researchers with an important tool when comparing different models, ultimately resulting in a stronger platform for making less arbitrary and potentially biased choices in terms of determining the number of profiles. Another weakness in previous studies is that the person-oriented analyses were based on single samples, raising the question of how robust and replicable the found cluster structures are. To address this latter problem, we draw data from two independent samples in the present study.

The purpose of the present study was to: (a) identify different motivational profiles, based on the different behavioural regulations for exercise in two samples of adults using latent profile analyses; and (b) examine differences in satisfaction of basic psychological needs (competence, autonomy and relatedness) and exercise behaviour across the different latent motivational profiles.

## Methods

### Participants

Sample A consisted of 1084 (279 men and 805 women) adults who were active members of an internet-based physical activity program created by a Swedish enterprise offering health care services in the private sector. The mean age was 45.0 years (*SD* = 11.7). Sample B comprised 511 (226 men and 285 women) university students with a mean age of 22.0 years (SD: 3.3). In sample A, mean levels of activity were 3.7 light exercise (*SD* = 3.3), 3.5 moderate exercise (*SD* = 2.9) and 1.9 strenuous exercise (*SD* = 1.7). The numbers for each exercise category represent number of times in an average week that participants did exercise on that specific intensity level for more than 15 min each occasion. Calculated and transformed into metabolic equivalent (MET) scores, sample A had an average expenditure of 44.2 (*SD* = 25.1) METS. For sample B, the mean levels were 2.9 (SD = 2.0) for light, 2.4 (SD = 2.7) for moderate and 2.3 (SD = 2.0) for strenuous exercise. The total MET score for sample B was 41.0 (SD = 26.0). The samples were thus roughly comparable in terms of exercise behaviour.

### Measures

In both samples A and B behavioral regulations were measured by the Behavioural Regulation in Exercise Questionnaire-2 (BREQ-2; [32]). The BREQ-2 comprised 19 items and five factors: amotivation (e.g., *“I don’t see the point in exercising”*), extrinsic regulation (e.g., *“I exercise because other people say I should”*), introjected (e.g., *“I feel guilty when I don’t exercise”*), identified (e.g., *“It’s important to me to exercise regularly”*) and intrinsic motivation (e.g., *“I exercise because it’s fun”*). The original BREQ-2 employed a five-point Likert-type scale ranging from 0 to 4, where 0 = “*not true for me*” and 4 = “*very true for me*”. In sample A, a four-point scale ranging from 1 to 4 but with the same anchors was used (1 = “*not true for me*” and 4 = “*very true for me*”), whereas the original scale from 0 to 4 was used in sample B. The use of a four point scale in sample A was due to the online survey design. Despite the different scale used from the original version, this four point scale version has been found to be psychometrically sound in the sample used in the present study [33]. In the present studies Cronbach’s alphas for the BREQ-2 subscales ranged from .73 to .92.

Basic psychological needs were measured with the Basic Psychological Needs in Exercise Scale (BPNES; [34]). The BPNES measures satisfaction of the needs for autonomy (e.g., *“I feel that the way I exercise is the way I want to”*), competence (e.g., “*I feel exercise is an activity which I do very well*”) and relatedness (e.g., “*My relationships with the people I exercise with are close*”), through 12 items employing a five point Likert-type scale, where 1 =”I don’t agree at all”and5 = ”I completely agree”. In the present studies Cronbach’s alphas for the BPNES subscales ranged from .81 to .92.

Self-reported exercise behaviour was assessed by the Godin Leisure-Time Exercise Questionnaire (LTEQ; [35]). Respondents indicate the frequency of mild, moderate, and strenuous exercise undertaken in a typical week. These scores are weighted by approximate metabolic equivalents for the different levels of activity (3, 5, and 9 respectively) and summed to produce an overall weekly physical activity score. Studies have shown the LTEQ to have adequate reliability and validity with respect to objective assessments of exercise behaviour and indices of fitness [36].

### Procedures

The BREQ-2, BPNES, and LTEQ were translated from English to Swedish according to the Back-Translation-method [37]. A bilingual (English and Swedish) expert first translated the tests from English to Swedish, and then another bilingual expert translated the tests back to English. Differences in the translated versions and the originals were discussed in the research group and formed the foundation of the final versions. The Swedish versions of these instruments have been found to be psychometrically sound [33]. Collection of sample A data was initiated by a pilot study of ten persons selected by convenience sample in order to test the comprehension and design of the test battery, using the Think Aloud-method [38]. Sample A completed the survey online in a research project initiated by XX XX. The participants were contacted by e-mail which was retrieved from a list of members provided by the health care service company, informing the participants of the aim of the study, ethical concerns and practical issues (e.g., the link to the web based survey). The collected data were stored at a web account only accessible by the researchers. No personal data were asked for; hence no personal register was created. The study was approved by the regional ethical board. For Sample B, the study was also initiated with a pilot study similar to sample A, including twenty participants. The data were collected during the students’ classes, at two different universities in the south of Sweden. The participants were informed about the aim of the study and ethical concerns prior to filling out the questionnaire, and provided informed consent. The study was approved by the regional ethical board (Dnr. Etik:H15 2010/94).

### Analyses

Descriptive statistics were obtained using SPSS version 20. Mplus software (version 7.1;[39]) was used to perform latent profile analysis (LPA). Model parameters were calculated using maximum likelihood (ML) estimation. Latent profile analysis was performed with the five BREQ-2 subscales (amotivation, external regulation, introjected regulation, identified regulation and intrinsic motivation) as input variables. A sequence of nested models, with an increasingly number of profiles, starting with one, where compared to examine if more complex models (with more profiles) fit the data better than more parsimonious models with less profiles. In the present study, models with one to seven profiles were tested to identify the optimal number of profiles. Profiles were added iteratively to identify the best model fit. Based on recommendations from previous research [40] a number of different criteria were used to determine the optimal number of profiles. The log likelihood (LL), the Bayesian Information Criterion (BIC; [41] and the sample-size adjusted BIC [42] were inspected, with lower values indicating better model fit. The Lo-Mendell-Rubin likelihood test (LMR: [43] and the Bootstrapped Likelihood Ratio Test (BLRT; [44] were used to compare the fit of two competing models. Statistically significant LMR and BLRT tests (*p* < .05) indicate that the target profile solution fits better with the data than a profile solution with one less profile. The entropy criterion was also examined, which indicates how accurately people are profiled into their respective profiles, with higher values indicating a better fit for a given solution [45]. In addition to the fit criteria, interpretability, theoretical meaningfulness and parsimony was also taken into account when deciding upon the best solution. Consequently, when merging the total information together for the final choice of model, subjective dimensions of choice beyond fit statistics were necessarily involved. More parsimonious models with fewer profiles were chosen over more complex profiles where this enhanced the interpretability of the profiles. To support the interpretation of the best-fitting solution, z-scores of the observed variables (i.e., the five BREQ-2 subscales) were used. To examine how the different latent profiles differed in terms of other relevant variables, the three basic psychological needs and exercise behavior were included in the model as auxiliary variables [46]. Mplus computes an overall test of association using Wald’s test as well as pairwise profile comparisons between the auxiliary variable means and probabilities. To clarify the LPA models used, the five BREQ-2 variables were used as predictors of the latent motivational profiles, whereas basic needs and exercise behaviours were outcome variables.

## Results

Descriptive statistics and correlations between the BREQ-2 subscales in Samples A and B are described in Table 1.

**Table 1:** Descriptive statistics and correlations between BREQ-2 variables in samples A and B

Variables	M	SD	1	2	3	4
Sample A^1^						
1. Amotivation	1.09	0.26	-			
2. External regulation	1.16	0.35	.17**	-		
3. Introjected regulation	2.17	0.77	-.14**	.23**	-	
4. Identified regulation	3.21	0.64	-.37**	-.15**	.35**	-
5. Intrinsic motivation	3.28	0.69	-.35**	-.19**	.07*	.70**
Sample B^2^						
1. Amotivation	0.33	0.62	-			
2. External regulation	0.40	0.58	.06	-		
3 .Introjected regulation	1.81	1.01	-.25**	.28**	-	
4. Identified regulation	2.84	0.90	-.54**	.01	.49**	-
5. Intrinsic motivation	2.93	0.92	-.48**	-.13**	.26**	.73**

*Note.* ^1^ *N* = 1089; ^2^ *N* = 511

BREQ-2 variable scores range from 1–4 in sample A and from 0–4 in sample B

*p < .05; **p < .01

### Identifying the optimal number of profiles for motivational type

A number of models, with increasing number of latent profiles, were tested and compared to identify the model that fit data best, both from a statistical as well as from a substantive point of view. This procedure was conducted independently in the two samples. The fit indices of the different models tested are described in Table 2. Lower values for LL, BIC and SSA-BIC, higher values for entropy and significant LMR and BLRT tests indicate better model fit compared with models including fewer profiles. In general, LL, BIC and SSA-BIC decreased for each model (being lowest in the 7-profile model), indicating a constant improvement of the model as additional profiles were modelled. The entropy indices generally increased from 2 to 3 profiles in both samples but then stabilized as additional profiles were added and consequently gave no clear indication of which model to choose. The BLRT indicated that each model fitted data better than a K-1 profile model, again pointing to the choice of the 7-profile model. However, other indices indicated that a model with fewer profiles than 7 would be a better choice. According to the Adjusted Lo-Mendell-Rubin likelihood ratio test (LMR), 2-3-4-5 and 6 profile models fitted the data better compared to a profile with one less profile (k-1 profile model) for both samples. However, the 7-profile models did not fit significantly better compared to 6 profile models, pointing to the notion that a 6-profile model would be the most suitable choice in both samples.

**Table 2:** Fit indices, entropy, and model comparisons for estimated latent profile analyses models in samples A and B

Model	LL	BIC	SSA-BIC	Entr	LMR	BLRT	nC < 10/5%
Sample A							
1 profile	−7493.75	15057.43	15025.67	-	-	-	-
2 profile	−6685.72	13462.34	13421.05	0.75	<.001	<.001	0/0
3 profile	−6303.14	12746.15	12682.62	0.81	>.05	<.001	0/0
4 profile	−5935.00	12058.81	11973.05	0.83	<.001	<.001	0/0
5 profile	−5755.10	11747.97	11639.98	0.85	<.001	<.001	0/0
** 6 profile**	**−5592.75**	**11472.22**	**11472.223**	**0.85**	**<.05**	**<.001**	**1/0**
7 profile	−5490.52	11316.71	11164.25	0.86	>.05	<.001	2/0
Sample B							
1 profile	−3622.88	7283.18	7264.14	-	-	-	-
2 profile	−3290.39	6661.85	6620.58	0.74	<.01	<.001	0/0
3 profile	−3178.18	6481.09	6417.61	0.83	<.01	<.001	0/0
4 profile	−3062.57	6293.52	6207.82	0.79	<.05	<.001	0/0
5 profile	−2936.11	6084.27	5976.35	0.84	<.001	<.001	0/0
** 6 profile**	**−2876.71**	**6009.11**	**5878.97**	**0.86**	**<.05**	**<.001**	**0/1**
7 profile	−2828.75	5956.86	5804.50	0.84	>.05	<.001	1/1

*Note.* Boldface rows describe the best fitting model in both samples

*LL* Log-likelihood, *BIC* Bayesian Information Criterion, *SSA-BIC* Sample Size Adjusted Bayesian Information Criterion, *LMR* *p*-value for Adjusted Lo-Mendell-Rubin likelihood ratio test, *BLRT* *p*-value for bootstrap likelihood ratio test

nC < 10/5% = number of profiles with less than 10 and 5% of the cases respectively

Aside from the statistical fit indices, the 5–6 and 7-profile model solutions were also closely inspected to identify the substantive and theoretical meaningfulness of respective solutions. The result of this examination was that the 6-profile model was interpreted to constitute the most substantive, theoretically meaningful and parsimonious solution. In light of the fact that both statistical as well as substantive arguments favored the 6-profile model, we choose to move forward with these models in subsequent analyses in both samples.

### Interpretation of the best fitting six-profile solutions

**Fig. 1:**
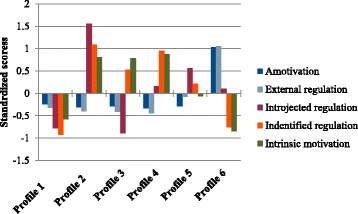
Motivational profiles in best fitting model (6 profiles) in sample A

**Fig. 2:**
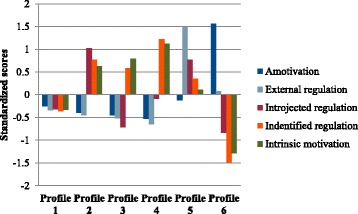
Motivational profiles in best fitting model (6 profiles) in sample B

A description of the different profiles of the 6-profile solutions in Samples A and B is found in Table 3 and also graphically illustrated in Fig. 1 (Sample A) and Fig. 2 (Sample B). The coefficients included in Table 3 and situated on the Y-axis of Figs. 1 and 2 are z-scores (standardized scores). Negative scores means that individuals in this profile scored lower than the average (of that sample), positive scores means that individuals scored higher than the average (of that sample) and scores around 0 means that individuals reported average scores. In Sample A, profile 1 (*n* = 194, 17.8%) is characterized by low scores on all variables, in particular on introjected regulation (z = − 0.78, *p* < .01) and identified regulation (z = − 0.92, *p* < .01). Consequently profile 1 may be labeled a “*low motivation profile*”. In contrast to profile 1, individuals in profile 2 (*n* = 87, 8.0%) report very high levels of introjected regulation (z = 1.56, *p* < .01) identified regulation (z = 1.09, *p*<.01) and intrinsic motivation (z = 0.81, *p*<.01). Consequently, this profile seems to be a “*self-determined and introjected profile*”. Profile 3 (*n* = 200, 18.4%), resembles profile 2 in terms of low scores on amotivation (z = − 0.29, *p* < .01) and external regulation (z = − 0.41, *p* < .01), and high scores on intrinsic motivation (z = 0.79, *p* < .01). However, in contrast to profile 2, profile 3 is characterized by lower scores on identified regulation (z = 0.53, *p* < .01). More importantly, contrary to the high scores on introjected regulation for individuals in profile 2, individuals in profile 3 report very low scores on introjected regulation (z = − 0.89, *p* < .01). Profile 3 is therefore labelled a *“self-determined and low introjected profile”*. Similar to profiles 2 and 3, individuals in profile 4 (*n* = 115, 10.5%) show below average amotivation (z = − 0.33, *p* < .01 and external regulation (z = − 0.44, *p* < .01), and almost a standard deviation above the mean in identified regulation (z = 0.95, *p* < .01 and intrinsic motivation (z = 0.87, *p* < .01). However, in contrast to profiles 2 and 3, the average levels of introjected regulation are not high or low but on average levels (z = 0.16, *p* < .01). Overall profile 4 mirrors a “*self-determined profile*”. Individuals in profile 5 (*n* = 263, 24.1%) depict a quite different motivational pattern. This profile is primarily characterized by a relatively high introjected regulation (z = 0.56, *p* < .01) in combination with slightly above mean scores on identified regulation (z = 0.21, *p* < .01) and slightly below mean scores on amotivation (z = − 0.29, *p* < .01). This profile is named an “*introjected and identified motivation profile*”. Finally, individuals in profile 6 show high scores on amotivation (z = 1.03, *p* < .01) and external regulation (z = 1.05, *p* < .01) but low scores on identified regulation (z = − 0.76, *p* < .01) and intrinsic motivation (z = − 0.85, *p* < .01). Profile 6 is consequently labelled an “*amotivated and controlled motivation profile*”.

**Table 3:** Description of the six latent profiles based on standardized BREQ-2 variables for samples A and B

BREQ-II- variables	Profile1	Profile2	Profile3	Profile4	Profile5	Profile6
Sample A						
Amot	−0.24*	−0.31*	−0.29*	−0.33*	−0.29*	1.03*
Ext reg	−0.32*	−0.40*	−0.41*	−0.44*	−0.08	1.05*
Introj reg	−0.78*	1.56*	−0.89*	0.16*	0.56*	0.10
Ident reg	−0.92*	1.09*	0.53*	0.95*	0.21*	−0.76*
Intrins mot	−0.58*	0.81*	0.79*	0.87*	−0.06	−0.85*
Sample B						
Amot	−0.25*	−0.40*	−0.45*	−0.53*	−0.12	1.56*
Ext reg	−0.34*	−0.45*	−0.52*	−0.65*	1.50*	0.08
Introj reg	−0.32*	1.03*	−0.71*	−0.09	0.77*	−0.84*
Ident reg	−0.36*	0.77*	0.58*	1.22*	0.35*	−1.49*
Intrins mot	−0.33*	0.63*	0.79*	1.12*	0.11	−1.28*

*Note:* *p < .01

Sample A: Profile 1 = *low motivation* (*n* = 194,17.8%); Profile 2 = *self-determined with high introjected regulation* (*n* = 87, 8.0%); Profile 3 = *self-determined with low introjected regulation* (*n* = 200, 18.4%); Profile 4 = *self-determined motivation* (*n* = 115, 10.5%); Profile 5 = *introjected and identified regulation* (*n* = 263, 24.1%); Profile 6 *=* amotivated and controlled motivation (*n* = 230, 21.1%); Sample B : Profile 1 = *low motivation* (*n* = 140, 27.4%); Profile 2 = *self-determined and high introjected regulation* (*n* = 101, 19.8%); Profile 3 = *self-determined and low introjected* (*n* = 75, 14.7%); Profile 4 = *self-determined motivation* (*n* = 21, 4.1%); Profile 5 = *extrinsic motivation* (*n* = 90,17.6%); Profile 6 = *amotivated* (*n* = 84, 16.4%)

In sample B, profile 1 (*n* = 140, 27.4%) has below average scores on all variables, thus being a “*low motivation profile*”. Individuals in profile 2 (*n* = 101, 19.8%) show low scores on amotivation (z = − 0.40, *p* < .01) and external regulation (z = − 0.45, *p* < .01) in combination with high scores on identified regulation (z = 0.77, *p* < .01) and intrinsic motivation (z = 0.63, *p* < .01). They also show very high scores (z = 1.03, *p* < .01) on introjected regulation, taking the total profile of a *“self-determined and high introjected profile”*. Profile 3 (*n* = 75, 14.7%) is similar to profile 2 in its low scores on amotivation (z = − 0.45, *p* < .01), external regulation (z = − 0.52, *p* < .01) and above mean scores (albeit not as very high as in profile 2) on identified regulation (z = 0.58, *p* < .01), and intrinsic motivation (z = 0.79, *p* < .01). Profile 3 distinguishes itself from profile 2 with low scores on introjected regulation (z = − 0.71, *p* < .01). In general, profile 3 is labelled a “*self-determined and low introjected profile*”. The very small profile 4 (*n* = 21, 4.1%) is described by low levels of amotivation (z = − 0.53, *p* < .01) and external regulation (z = − 0.65, *p* < .01), average levels of introjected regulation (z = − 0.09, *p* > .05), and very high identified regulation (z = 1.22, *p* < .01) and intrinsic motivation (z = 1.12, *p* < .01). Overall, profile 4 could clearly be described as a “*self-determined profile*”. Individuals in profile 5 (*n* = 90, 17.6%), report average scores on amotivation and intrinsic motivation, but very high scores on external regulation (z = 1.50, *p* < .01), high scores on introjected regulation (z = 0.77, *p* < .01), and above mean scores on identified regulation (z = 0.35, *p* < .01). Consequently this profile seems to be an “*extrinsic motivation profile*”. Profile 6 (*n* = 84, 16,4%), is labelled an “*amotivated profile*” with very high scores on amotivation (z = 1.56, *p* < .01) and low to very low scores on introjected regulation (z = − 0.84, *p* < .01), identified regulation (z = − 1.49, *p* < .01), and intrinsic motivation (z = − 1.28, *p* < .01).

### Differences in basic psychological needs, motivation regulations and exercise behavior across latent motivational profiles

Latent profile differences in basic need satisfaction and exercise behavior are shown in Table 4. In Sample A, the overall test of equality of means were statistically significant for competence χ^2^ (5) =124.06, *p* < .001, autonomy χ^2^ (5) =69.18, *p* < .001, relatedness χ^2^ (5) =55.84, *p* < .001, and exercise behavior χ^2^ (5) =16.11, *p* < .01. Similar results were found in Sample B, with the overall equality test being significant for competence χ^2^ (5) =113.32, *p* < .001, autonomy χ^2^ (5) =101.04, *p* < .001, relatedness χ^2^ (5) =72.64, *p* < .001, and exercise behavior χ^2^ (5) =41.03, *p* < .01. More specifically, in Sample A, profiles 2,3 and 4 demonstrated higher satisfaction in terms of all three psychological needs (competence, autonomy and relatedness) compared to profiles 1, 5 and 6 (ps < .05). Moreover, profile 6 showed higher competence and relatedness satisfaction compared to profiles 1 and 5 and higher autonomy compared to profile 5 (ps < .05). In terms of exercise behavior, profiles 2–4 reported exercising more compared to profiles 1 and 5 (ps < .05) and profile 2 also reported more exercise compared to profile 6. Profile 6, however, exercised more compared to profiles 1 and 5.

**Table 4:** Differences across the six latent motivational profiles in psychological need satisfaction and exercise in both samples

Variables	Profile 1	Profile 2	Profile 3	Profile 4	Profile 5	Profile 6
	M (S.E.)	M (S.E.)	M (S.E.)	M (S.E.)	M (S.E.)	M (S.E.)
Sample A						
Comp	13.89^c^ (0.23)	17.17^a^ (0.17)	16.83^a^ (0.33)	17.55^a^ (0.21)	13.31^c^ (0.26)	14.93^b^ (0.19)
Auto	15.43^bc^ (0.25)	17.73^a^ (0.19)	17.53^a^ (0.36)	17.99^a^ (0.22)	14.90^c^ (0.25)	15.92^b^ (0.21)
Rel	14.02^c^ (0.34)	17.40^a^ (0.25)	16.41^a^ (0.46)	17.31^a^ (0.33)	13.25^c^ (0.32)	15.25^b^ (0.25)
Exercise	37.83^c^ (1.50)	52.58^a^ (2.54)	49.71^ab^ (2.64)	49.99^ab^ (2.02)	37.83^c^ (2.39)	45.12^b^ (1.54)
Sample B						
Comp	12.10^d^ (0.39)	14.70^b^ (0.53)	15.83^ab^ (0.53)	17.05^a^ (0.64)	13.31^c^ (0.45)	10.15^e^ (0.61)
Aut	12.94^d^ (0.41)	15.08^b^ (0.60)	16.57^ab^ (0.47)	17.97^a^ (0.59)	14.39^c^ (0.51)	11.51^e^ (0.63)
Rel	13.14^b^ (0.50)	16.19^a^ (0.47)	16.58^a^ (0.65)	17.26^a^ (1.24)	13.56^b^ (0.65)	10.87^c^ (0.69)
Exercise	36.75^b^ (2.51)	54.38^a^ (4.29)	51.30^ab^ (4.74)	58.22^a^ (8.00)	41.27^b^ (3.56)	26.62^c^ (3.75)

*Note:* *Comp*: Competence; *Aut*: Autonomy; *Rel*: Relatedness; Exercise: Total LTEQ score

^a,b,c,d,e^Values in the same row that do not share a common subscript are significantly different at *p* < .05

Description of profile labels: *Sample A*: Profile 1 = low motivation; Profile 2 = self-determined with high introjected regulation; Profile 3 = self-determined with low introjected regulation; Profile 4 = self-determined motivation; Profile 5 = introjected and identified regulation; Profile 6 = amotivated and controlled motivation; *Sample B* : Profile 1 = low motivation; Profile 2 = self-determined and high introjected regulation; Profile 3 = self-determined and low introjected; Profile 4 = self-determined motivation; Profile 5 = extrinsic motivation; Profile 6 = amotivated

In Sample B, profiles 2, 3, and 4 reported higher need satisfaction in terms of competence, autonomy and relatedness compared to profiles 1, 5 and 6 (ps < .05). Profiles 2 and 4 also revealed higher exercise scores compared to profiles 1, 5 and 6.

Furthermore, profile 5 had higher competence and autonomy scores compared to profiles 1 and 6 and profile 1 reported higher competence and autonomy compared to profile 6 (ps < .05). Profiles 1 and 5 also revealed higher scores on relatedness and exercise more compared to profile 6 (ps < .05).

## Discussion

The aim of the study was to identify latent profiles of motivation for exercise, using a person-centred analytical approach, and to compare these motivation profiles in terms of basic psychological needs and exercise behaviour. In both samples, six distinct profiles of motivational regulations in exercise were found. Three broad questions to discuss are how well these profiles (a) replicate across samples; (b) match the profiles found in previous work linked to physical activity; and (c) are aligned with the SDT and OIT theories.

Starting with the first question, two different samples, with different characteristics and exercise contexts were used in the study. Individuals in sample A were middle-aged adults taking active part in an online exercise program, whereas sample B comprised undergraduate students not involved in any particular program. Consequently, there were reasons to expect that the overall patterns of profiles found would not be identical across the samples. On the other hand, given the assumed universality of the central tenets in OIT, some degree of replication across samples was also expected. In line with this reasoning, the results did show that some similar profiles were found in both samples whereas other profiles seemed to be more unique to each sample. In terms of similarity, primarily four types of profiles similar in shape were found across the two samples. The replicated four profiles include a low motivation profiles and three types of self-determined profiles differing primarily in introjection regulation; one with high, one with low, and finally one with average levels of introjection. Consequently, these four types of profiles seem to represent more stable and consistent subgroups of motivation regulations, at least in the present study.

Two other profiles in each sample (profiles 5 and 6) seem to be more unique across samples and less ubiquitous, such as the amotivated and controlled motivation profile in Sample A, or the extrinsic motivation profile (with above mean scores also on identified regulation) in Sample B. Such more unique profiles represent theoretically less easily explained interactions among regulations that nevertheless appear in data. These more untypical profiles may provide as important a theoretical insight as the more stable previously replicated profiles, as they demonstrate the complex result of within-person effects of different regulations interacting. From a broader perspective, these profiles also mirror the richness of the phenomenon of motivation, with multiple ways different driving forces and types of motivation can manifest themselves.

Linking the results with previous work, groups (clusters) similar to the self-determined profiles have been identified and discussed in studies in the field of physical activity [27–29], despite the fact that these studies used different (less active) samples compared to the present one. Given the theoretically robust underpinning of such a profiles, however, it is not unexpected that they replicate across samples and studies. The self-determined profile with high introjection has however not been so clearly identified in previous work, although clusters with high autonomous motivation in combination with moderate introjection have been noted [27–29]. In general, introjected regulation seems to play a special role in the current analyses, as it constituted the most obvious difference between some profiles otherwise quite similar in terms of the regulation patterns (profiles 2–4). This further highlights the complex role of introjected regulation from a classical SDT-related autonomous vs. controlled motivation division. It also points to issues raised in recent papers [13–15] regarding problems with the OIT continuum, where introjected regulation is theoretically labelled as a non self-determined (controlled) type of motivation despite the fact that it, at least empirically, seems to be more in the middle of controlled and self-determined motivation. In the present study, the variable-centered analyses clearly demonstrated that introjected regulation was moderately and positively associated with identified regulation in both samples. From a strict SDT and OIT perspective, these results may be unexpected, but they are in line with previous work showing that introjected regulation can accompany self-determined motivation [47]. A review of previous studies [9] indicated that the association between introjected regulation and physical activity was mixed and inconsistent, with the majority of studies showing either positive (30% of studies) or no (60% of studies) association. In the present study, the self-determined and high introjected profile in Sample A did show similarly high need satisfaction and exercise scores as the self-determined profiles with low or moderate introjection. Also in Sample B, this combined autonomous and introjection profile demonstrated similarly high exercise scores, significantly higher than other profiles, as the self-determined profiles with low or moderate introjection. This suggests that high introjected regulation, if combined with/ supported by, high autonomous motivation, may not be detrimental in terms of need satisfaction and exercise behaviour. Future studies should further examine how introjected regulation interacts with other types of motivation and its association with exercise behaviour, both from a variable-and person-centred perspective. A relevant question for future studies is also if the observed motivational patterns and profiles observed in the present study are unique for exercise or generalise to other health behaviors.

Viewing the pattern of profiles from a SDT and OIT related theoretical perspective, if the assumed qualitative differences between controlled and autonomous motivation really are important, one should in a person-centred analysis find, at least, these two opposing motivational profiles. In both samples, there were clear examples of self-determined, or autonomous profiles, combining low scores on external and introjected regulation with high scores on identified and intrinsic motivation. Support for this type of “beneficial” or high quality motivation profile was thus clear in the present study. On the other hand, there was less clear support for pure controlled motivation profiles, at least if such profiles are more tightly labelled as low scores on intrinsic, and identified in combination with high scores on both external and introjected regulation. The low motivation profiles, constituting of 18 and 27% of individuals in the two samples, are difficult to interpret and explain, not least given the lower than average scores on all regulations, including amotivation. One possible way to explain this profile is response style artifact. For example, for some individuals, none of the items in the scale may seem relevant or meaningful if these individuals are “exercise aschematics” [48], and do not process information linked to exercise the same way.

Despite the fact that the profiles found are somewhat different than the ones typically used in the original continuum, it could still be argued that they can be ordered along a continuum from highest to lowest motivation quality. Closest to the optimal motivational quality side of the continuum would be the self-determined profile with low introjection followed by the self-determined profile and the self-determined profile with high introjection. More in the middle would be the introjected and identified profile in sample A and the extrinsic profile in sample B. In the opposite end, on the lowest quality side, would be the amotivated and controlled profile in sample A and the amotivated profile in sample B along with the two low motivation profiles.

The identification of the new interaction and- within-person based motivational profiles in the present study may also shed new light on the discussion [49–51] concerning the relationship between, and integration of, SDT and motivational interviewing (MI). For example, the notion of profiles consisting of different co-existing regulations that span across both controlled and autonomous motivation (e.g., introjection regulation operating in conjunction with the self-determined sources of identified regulation and intrinsic motivation) open up for questions for whom (for what specific motivational profiles) and when (under what circumstances) certain MI constructs and techniques are most effective in creating a sustained driving force for behaviour change. Perhaps profiles such as the ones identified in the present study (self-determined motivation coupled with introjection regulation) allow SDT to better incorporate types of motivation-related concepts such as ambivalence and resistance.

From a broader methodological perspective, the present study adds to the list of recent papers [13–15] highlighting contradictions and problems associated with the assumptions underlying the OIT and how motivation is conceptualised and operationalised through the unidimensional continuum. Although we have used a different analytical lens compared to these studies, the results of the present study go in the same direction as these studies and support the notion that motivation should best be conceptualized as multidimensional [15], varying in kinds, types or qualities rather than degree of relative autonomy [14]. Also, the results of the person-oriented analyses in the present study further highlight the importance and value of going beyond simple effects of separate motivational constructs and regulations [15] to elucidate interactions of different motivational forces, simultaneously pulling and pushing the individual closer to, or away from, certain behaviours.

As demonstrated by the different profiles, there are a number of individuals who display within-person interactions of multiple regulations that may not be neatly classified or interpreted according to a standard SDT or OIT framework (typically interpreting regulations as either autonomous or controlled). For example, some of the profiles found further highlight the complexity of the interplay between extrinsic regulations (external, introjected and identified regulations). According to the OIT and the regulation continuum, external and introjected regulations are supposed to tap controlled forms of motivation, whereas identified regulation is an autonomous form. However, the variable-centred analyses depicted a complex pattern of associations between these three regulations, with positive relations between on one hand external and introjected regulations, but on the other hand also between identified and introjected. Profile 5 in sample B illustrates that, some individuals may be driven by all three extrinsic regulations simultaneously, crossing over the standard SDT division into autonomous vs. controlled forms of motivation. Profiles 2 in both samples also provide examples of subgroups of individuals whose exercise behaviour is regulated both by more autonomous forms of motivation (intrinsic and identified) as well as introjected regulation.

From a construct validity point of view, an important issue is whether the profiles found are merely statistical artefacts, or if there is a meaningful pattern of associations between these profiles and other key variables. In general, results show that the profiles being characterised by high autonomous motivation also demonstrated higher need satisfaction compared to profiles with more mixed or controlled motivational patterns. Also, although less apparent, the more autonomously motivated profiles also demonstrated exercising more regularly, in particular compared with controlled motivation profiles. Taken together, these results add to the substantial body of previous work that has documented associations between autonomous motivation and positive outcomes such as higher need satisfaction and physical activity [9, 33]. The results are also in line with previous work (having also used person-centred analysis) demonstrating that participants in more self-determined profiles enjoy exercise more [28], are more physically active [29] and belong to later stages of change in terms of exercise, that is, are more sustainable in their behaviour [27].

Linked to the question of the potential value of the person-centered analyses is also a question of whether the results reflect only quantitative differences in levels between profiles, or also qualitative differences in terms of shape [30]. For example, if identified profiles would primarily differ in terms of their overall levels (e.g., one profile being equally higher or lower on all variables compared to another profile), the results of the LPA may not contribute much new information. However, in the present study, differences between profiles were foremost a question of shape rather than only overall levels. As such, differences between profiles were characterized by the differentiated interactional pattern of the different regulations, that is, the relative strength of regulations compared to other regulations within persons, rather than merely differences in terms of levels.

The cross-sectional design of the study is one apparent weakness of the present study. For example, no causal direction can be implied by the associations between the profiles, need satisfaction and exercise behaviour. Also, although the data displayed substantial variation terms of most the BREQ-2 input variables, there was very little variance, and low overall scores in amotivation, which may have provided a different overall pattern compared with if the samples also had included more people being less active and scoring high on amotivation. Finally, the reliance on self-report data for both the independent (motivation regulation) and dependent variables (e.g., needs and exercise behaviour) may have contributed to inflation of the associations found.

Translating the results to practical implications, the identification of distinct subgroups with different motivational patterns should be of high relevance to practitioners on the field, as this also may be an important starting point for developing more specific and tailored interventions aimed at getting individuals more physically active and maintaining a sustainable exercise regime across time. As suggested in previous work [28], practitioners could draw both on similarities and differences between the different profiles to develop sharper and more individually tailored programs that target multiple and simultaneously interacting motivational regulations at the same time. For example, the different profiles identified in the present study would likely not respond similarly to a general intervention program. Both the pathway from the intervention to the presumed mechanisms/mediators for change (action theory link; [52]) as well as the effect of the mediators on the outcome (conceptual theory link; [52]) may operate differently for subgroups with different motivational profiles.

## Conclusions

To conclude, as clearly indicated by the present results, a number of relevant within-person interactions between multiple regulations seem to exist, manifested in distinct motivational profiles. In other words, people have different and multiple (and often motivationally and theoretically conflicting) reasons for engaging in exercise behaviour. The motivational soup may therefore be hard to accurately describe through too rigid theoretically driven lenses, in particular if one uses traditional variable-centred analysis. These interactions, embodied as latent profiles, also seem to be related with other key variables, such as need satisfaction and exercise behaviour. Identifying such complex interactions, consisting of five interacting variables, would most probably be futile in traditional variable-centered analyses, and if identified statistically, the interpretation of these five-way interactions would be very cumbersome. As such, the value of using person-centered analysis, such as LPA, aside from providing a complementary and alternative picture of associations, may primarily be to offer researchers and practitioners the possibility to closer examine the important SDT-related assumption of multiple regulations/driving forces pushing and pulling the individual towards behaviour; a theoretical assumption that has been largely neglected, or only touched upon on the surface, in previous empirical work. Put differently, if we are to understand the different layers of the motivational soup, and more importantly how these layers interact within-persons to create the behavioural flavour of the motivational soup (i.e., the exercise behaviour), person-centred analysis may be a relevant way forward, as it can provide an alternative but informative picture of the complex motivational interactions and their role for exercise behaviour. A conclusion of the present study is that the motivational soup is likely blurry, complex and very heterogeneous for different groups, but patterns can be detected, if one looks close enough and uses suitable methodological and analytical glasses.
